# *Drosophila melanogaster* as a versatile model organism to study genetic epilepsies: An overview

**DOI:** 10.3389/fnmol.2023.1116000

**Published:** 2023-02-16

**Authors:** Florian P. Fischer, Robin A. Karge, Yvonne G. Weber, Henner Koch, Stefan Wolking, Aaron Voigt

**Affiliations:** ^1^Department of Epileptology and Neurology, RWTH Aachen University, Aachen, Germany; ^2^Department of Neurology and Epileptology, Hertie Institute for Clinical Brain Research, University of Tübingen, Tübingen, Germany; ^3^Department of Neurology, RWTH Aachen University, Aachen, Germany; ^4^JARA-BRAIN Institute Molecular Neuroscience and Neuroimaging, Forschungszentrum Jülich GmbH and RWTH Aachen University, Aachen, Germany

**Keywords:** *Drosophila melanogaster*, epilepsy, genetics, precision medicine, techniques, translational research

## Abstract

Epilepsy is one of the most prevalent neurological disorders, affecting more than 45 million people worldwide. Recent advances in genetic techniques, such as next-generation sequencing, have driven genetic discovery and increased our understanding of the molecular and cellular mechanisms behind many epilepsy syndromes. These insights prompt the development of personalized therapies tailored to the genetic characteristics of an individual patient. However, the surging number of novel genetic variants renders the interpretation of pathogenetic consequences and of potential therapeutic implications ever more challenging. Model organisms can help explore these aspects *in vivo*. In the last decades, rodent models have significantly contributed to our understanding of genetic epilepsies but their establishment is laborious, expensive, and time-consuming. Additional model organisms to investigate disease variants on a large scale would be desirable. The fruit fly *Drosophila melanogaster* has been used as a model organism in epilepsy research since the discovery of “bang-sensitive” mutants more than half a century ago. These flies respond to mechanical stimulation, such as a brief vortex, with stereotypic seizures and paralysis. Furthermore, the identification of seizure-suppressor mutations allows to pinpoint novel therapeutic targets. Gene editing techniques, such as CRISPR/Cas9, are a convenient way to generate flies carrying disease-associated variants. These flies can be screened for phenotypic and behavioral abnormalities, shifting of seizure thresholds, and response to anti-seizure medications and other substances. Moreover, modification of neuronal activity and seizure induction can be achieved using optogenetic tools. In combination with calcium and fluorescent imaging, functional alterations caused by mutations in epilepsy genes can be traced. Here, we review *Drosophila* as a versatile model organism to study genetic epilepsies, especially as 81% of human epilepsy genes have an orthologous gene in *Drosophila*. Furthermore, we discuss newly established analysis techniques that might be used to further unravel the pathophysiological aspects of genetic epilepsies.

## 1. Introduction

### 1.1. The principles of epilepsy

Epilepsy is one of the most frequent neurological disorders, with more than 45 million people affected worldwide (Beghi et al., 2019). It is characterized by an enduring predisposition to generate epileptic seizures that result from excessive or hypersynchronous neuronal activity in the brain (Fisher et al., 2014). The etiology of epilepsy is diverse, including structural, genetic, infectious, metabolic, immune as well as unknown causes (Scheffer et al., 2017). Current anti-seizure medications (ASMs) aim to achieve seizure control by suppression of seizure activity. Notwithstanding expansive research, medications that impact epileptogenesis or aim at etiologic factors are not in clinical use. Despite the availability of more than two dozen ASMs, approximately one-third of patients develop drug-resistant epilepsy, i.e., they display ongoing seizures. This leaves them at an increased risk of psychosocial dysfunction, reduced quality of life, and premature death (Loscher et al., 2020). Thus, there is an unmet clinical need to better understand the underlying disease mechanisms and to develop more effective, mechanistically driven therapies.

The last two decades have brought tremendous progress to epilepsy genetics. The advent of next-generation sequencing, e.g., targeted gene panels, whole exome and whole genome sequencing, as well as increasingly powerful bioinformatic tools have led to a surge in gene discovery for monogenic epilepsy syndromes (Moller et al., 2015). Many epilepsy genes encode ion channels, neurotransmitter receptors, solute carriers, synaptic vesicle proteins, transcription factors and proteins involved in metabolic pathways (Howard and Baraban, 2017). The growing knowledge of causative genetic variants spurred the search for personalized therapies that are tailored to a patient’s individual genetic characteristics. In fact, in a small subset of epilepsies, genetic findings have already been translated into effective therapies. Well-established examples are the ketogenic diet for GLUT1 deficiency syndrome (Kass et al., 2016) and vitamin B6 supplementation for *ALDH7A1*-related epilepsy (Coughlin et al., 2019). However, the discovery of genetic alterations raises the need to interpret their potential functional consequences - a prerequisite for the rational design of individualized treatments. Pathogenic variants within the same gene can produce remarkably different phenotypes. For instance, variants of *SCN1A* cause a wide spectrum of epilepsies, ranging from mild generalized epilepsy with febrile seizures plus (GEFS+) to severe, intractable epilepsies, such as Dravet syndrome (DS; Catterall et al., 2010; Escayg and Goldin, 2010). Determining functional effects, e.g., loss-of-function (LOF) vs. gain-of-function (GOF), bears immediate consequences for personalized therapy strategies as in *SCN2A* and *GRIN2A/B*-related epilepsies (Wolff et al., 2017; Krey et al., 2022). Model systems can help analyze the functional consequences of novel candidate variants *in vivo*.

**Figure 1:**
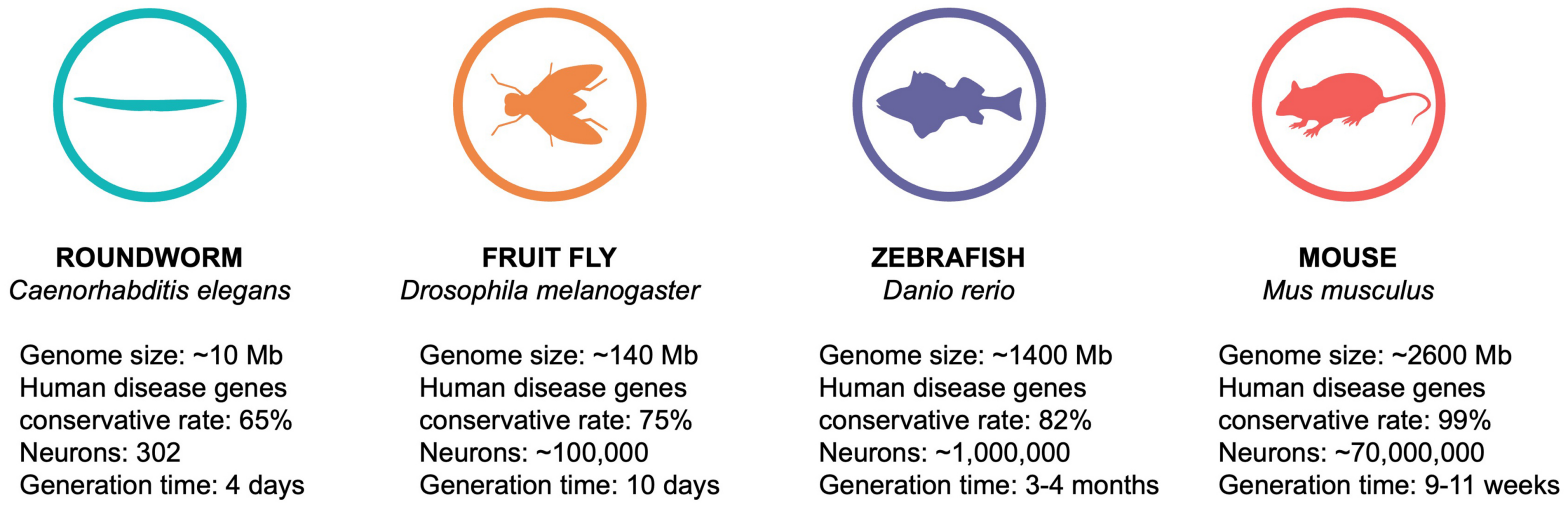
Comparison of animal models used in epilepsy research.

In the last decades, epilepsy has been studied in various animal models such as roundworms and zebrafish, but most traditionally in rodents (for comparison see Figure 1; Baraban, 2007; Cunliffe et al., 2015; Johan Arief et al., 2018; Takai et al., 2020; Marshall et al., 2021; Wang and Frankel, 2021). An optimal approach to address genotype–phenotype correlations would be to establish a library of transgenic mice expressing all identified candidate variants. However, such an approach would not only be extremely laborious, but also expensive and time-consuming. Therefore, additional model organisms that allow for large-scale functional studies in a reasonable time frame and in a cost-effective manner are urgently needed. The fruit fly *Drosophila melanogaster* has emerged as an increasingly attractive model system due to its small size, fast generation time, low maintenance costs and ease of genetic manipulation (Rosch et al., 2019). Recent developments in genome editing techniques have facilitated the generation and characterization of humanized flies, carrying the human epilepsy-causing mutation in the corresponding fly gene (Sun et al., 2012; Schutte et al., 2014; Roemmich et al., 2021). These disease models offer a unique opportunity to unravel the molecular mechanisms underlying genetic epilepsies and to explore potential therapeutic targets. In addition, *Drosophila* is a well-established model for high-throughput drug screening (Stilwell et al., 2006), eventually providing a rapid and inexpensive platform for the development of novel precision medicine therapies.

### 1.2. The basics of fruit flies

The fruit fly *Drosophila melanogaster* has been widely used as a genetic model organism in biomedical research for more than 100 years. It has greatly advanced our understanding of a broad range of biological processes including genetics, inheritance, embryonic development, learning, and behavior (Jennings, 2011). There are many advantages that make *Drosophila* an attractive model organism, e.g., the ease of maintenance, cost-effectiveness, fewer ethical restrictions, and the availability of a large and sophisticated genetic toolbox. The genome of *Drosophila* has been completely sequenced. It comprises around 13,600 protein coding genes that are distributed on four chromosomes (Adams et al., 2000). Approximately 75% of all human disease-related genes have an orthologue in the fly, making it a valuable model organism to study human diseases (Reiter et al., 2001). The short life cycle (10 days at 25°C) of *Drosophila* is composed of four developmental stages: embryo, larva, pupa, and adult. In addition to the short generation time, the large number of offspring facilitates statistical analysis. The adult fly brain comprises about 100,000 neurons that form discrete circuits that are responsible for complex behaviors such as courtship, sleep, circadian rhythms, learning, and memory (Pandey and Nichols, 2011). Although the anatomic structure of the fly brain differs considerably from that of the human brain, many fundamental functions of neurons, e.g., membrane excitability, voltage-gated ion channels, and neurotransmitter receptors, are highly conserved between the two species (Parker et al., 2011a).

One of the most striking advantages of the fly model is the availability of a large and sophisticated repertoire of genetic tools (reviewed in Hales et al., 2015). For instance, chemical agents, e.g., ethyl methyl sulfonate, or X-ray radiation can be leveraged to introduce random mutations into the genome of the fly. The resulting mutagenized flies can then be examined for a behavioral phenotype of interest. Such forward genetic screens are a suitable method to explore diseases, whose genetic underpinnings have not been elucidated. Conversely, a particular gene of interest can be evaluated for its phenotypic functions by RNA interference (RNAi)-mediated knock-down or gene overexpression. In addition, numerous genetic tools are available to facilitate precise genome editing, including transposable P-elements, homologous recombination, and CRISPR/Cas9. These techniques can be readily used to investigate the disease causality of rare variants found in human patients. A common approach is to knock-out or knock-down the *Drosophila* orthologue of the respective human gene and to analyze the resulting phenotype. If a phenotypic alteration is observed, wild-type and variant human cDNA are subsequently expressed. The causative nature of a variant may be confirmed if the observed phenotype is ameliorated by the wild-type but not the variant human cDNA (Wangler et al., 2015; Yamaguchi and Yoshida, 2018). All these aspects and techniques positioned *Drosophila* as a powerful model organism that is studied in a broad range of human diseases including Parkinson’s disease, Alzheimer’s disease, Huntington’s disease, amyotrophic lateral sclerosis, brain tumors, and epilepsy (Jeibmann and Paulus, 2009; Lenz et al., 2013; Prüssing et al., 2013).

## 2. *Drosophila* in epilepsy research

### 2.1. The class of bang-sensitive mutants

*Drosophila* has been used in epilepsy research since the discovery of the group of bang-sensitive mutants more than 50 years ago (Benzer, 1971). These flies typically respond with seizure-like behavior and paralysis to a mechanical shock (termed “bang”), such as a tap of the culture vial on the bench top or a brief vortex mixing (Song and Tanouye, 2008; Parker et al., 2011a; Burg and Wu, 2012). This complex behavioral phenotype can be divided into six distinguishable stages: (1) a shock-induced “initial seizure” that lasts several seconds and that is characterized by extensive wing flapping, leg shaking, abdominal contractions, and proboscis extensions; (2) a post seizure “paralysis” where the flies are completely immobile and do not respond to mechanical stimulation; (3) a “tonic–clonic phase” where the paralytic behavior is interrupted by multiple bouts of clonus-like activity (only observed in a fraction of bang-sensitive flies); (4) a “recovery seizure” that resembles the initial seizure and clonus-like activity; (5) a “refractory period” during which the flies exhibit normal behavior but are resistant to further seizure induction; and (6) a complete “recovery” where the flies re-acquire their bang-sensitivity. In addition to mechanical stimulation, the seizure-like phenotype can also be induced by high frequency electrical stimulation directly delivered to the fly brain. Each genotype has a specific seizure threshold at which seizure-like behavior occurs. Even wild-type flies will display seizure-like behavior if the voltage is high enough. However, the seizure threshold of bang-sensitive mutants is significantly lower than that of wild-type flies (Kuebler and Tanouye, 2000; Howlett and Tanouye, 2009). The bang-sensitive phenotype can be attenuated by treatment with several ASMs, e.g., valproate, phenytoin, gabapentin, and potassium bromide (Kuebler and Tanouye, 2002; Reynolds et al., 2003; Tan et al., 2004). In addition, mutations in specific genes can (partially) revert the behavioral phenotype of bang-sensitive mutants. Prominent examples of seizure-suppressor mutations include alleles of *Shaker* (*Sh*) and *shaking B* (*shakB*), which encode a potassium channel and a gap junction protein, respectively (Song and Tanouye, 2008). The identification of such seizure-suppressor mutations is challenging in humans but a well-established practice in *Drosophila*.

Since human epilepsy syndromes are often caused by mutations in genes encoding voltage-gated sodium channels (Escayg and Goldin, 2010; Johannesen et al., 2019), it is not surprising that a particular member of the bang-sensitive mutant class, i.e., *bang senseless* (*para^bss1^*), was found to carry a gain-of-function mutation in the *paralytic* (*para*) gene (Parker et al., 2011b). It encodes the only *Drosophila* voltage-gated sodium channel α-subunit and is orthologous to *SCN1A* to *SCN5A* and *SCN7A* to *SCN11A* in humans (Takai et al., 2020). The phenotype of *para^bss1^* is caused by a single amino acid substitution at position 1,699 (i.e., L1699F), which is located within the “paddle motif” of homology domain IV. Electrophysiology experiments in *Xenopus* oocytes showed that the mutation leads to a shift in voltage dependence of fast inactivation to more positive potentials (Parker et al., 2011b). This is consistent with the hypothesis that the voltage sensor paddle of homology domain IV is crucial for channel inactivation (Alabi et al., 2007; Bosmans et al., 2008). Thus, the L1699F variant renders neurons expressing *para^bss1^* more excitable and flies more prone to produce seizures (Parker et al., 2011b). The *para^bss1^* mutant is characterized by the most severe phenotype and the lowest seizure threshold of all bang-sensitive mutants. In addition, the phenotype of *para^bss1^* is the most difficult to suppress by ASMs or seizure-suppressor mutations, making it a suitable model for intractable epilepsy (Kroll et al., 2015a).

Another member of the bang-sensitive mutant class has been shown to carry a frame-shift mutation in the *easily shocked* (*eas*) gene, which encodes ethanolamine kinase, an enzyme involved in the synthesis of the membrane lipid phosphatidylethanolamine. The mutation results in a truncated protein that lacks enzymatic activity. It has been suggested that the bang-sensitive phenotype is caused by an altered membrane phospholipid composition (Pavlidis et al., 1994). Furthermore, there are several bang-sensitive mutants with impaired mitochondrial function. The affected genes include *technical knockout* (*tko*), *stress-sensitive B* (*sesB*), and *knockdown* (*kdn*), which encode a mitochondrial riboprotein, an ATP translocase, and citrate synthase, respectively (Royden et al., 1987; Zhang et al., 1999; Fergestad et al., 2006). ATP levels in these mutants are decreased, nurturing the hypothesis that metabolic perturbations may alter neuronal activity and increase seizure susceptibility (Fergestad et al., 2006). The underlying mechanism could be the impaired ability to maintain ionic gradients across the plasma membrane since the Na^+^/K^+^ ATPase is a large consumer of neuronal ATP. This is consistent with the observation that a specific mutation in the Na^+^/K^+^ ATPase α-subunit gene (i.e., the 2206 mutation) also results in a mild bang-sensitive phenotype (Schubiger et al., 1994; Pavlidis and Tanouye, 1995).

### 2.2. Other *Drosophila* mutants in epilepsy research

Besides bang-sensitive mutants, several other classes of *Drosophila* mutants have been used in epilepsy research. A prominent example is the group of temperature-sensitive paralytic mutants, which typically exhibit behavioral paralysis at elevated temperatures. A well-known member of this group is the *maleless* (*mle*) allele called *no-action potential temperature-sensitive* (*mle^napts^*; Song and Tanouye, 2008). Interestingly, adult *mle^napts^* flies display a reduction of voltage-gated sodium channels in their brains (Kauvar, 1982; Reenan et al., 2000). Importantly, *mle^napts^* has been shown to suppress seizures in bang-sensitive mutants in homozygous double-mutant condition (Kuebler et al., 2001). Another example of a temperature-sensitive paralytic mutant is *para^ST76^*, which carries a loss-of-function mutation in the *Drosophila* sodium channel gene *para* (Parker et al., 2011a). Like *mle^napts^*, this variant acts as a seizure suppressor for bang-sensitive mutants (Kuebler et al., 2001).

Another class of *Drosophila* mutants that have been used in epilepsy research are the so-called leg-shaking mutants. Prominent examples of this group include alleles of *shaker* (*sh*) and *ether a go-go* (*eag*), which encode different types of potassium channel subunits. Mutants of these genes display rapid leg-shaking in response to ether anesthesia, have heightened metabolic rates, and reduced life spans (Wang et al., 2000). Although the leg-shaking phenotype shares some similarities with the bang-sensitive phenotype, several mutants of these genes have been shown to have higher seizure thresholds than wild-type flies. This is in clear contrast to the bang-sensitive mutants, which typically display reduced seizure thresholds (Kuebler et al., 2001).

### 2.3. Modelling human *SCN1A*-related epilepsies in *Drosophila*

Mutations in the human *SCN1A* gene are known to cause GEFS+ and DS (Catterall et al., 2010), which are both autosomal dominant disorders. GEFS+ is a familial epilepsy syndrome that comprises a wide spectrum of clinical phenotypes. Affected individuals within GEFS+ families most commonly show febrile seizures that may persist beyond the age of 6 years. Other seizure types, such as absence, myoclonic, atonic, or focal seizures, may also be observed (Scheffer and Berkovic, 1997; Singh et al., 1999). Seizures are usually well controlled with ASMs (Catterall et al., 2010). In contrast, DS is a severe form of genetic epilepsy characterized by prolonged, febrile and afebrile, hemiclonic or generalized clonic seizures with onset in the first year of life. Over time, other seizure types appear including myoclonic and atypical absence seizures. Seizures are often refractory to treatment and patients display several other clinical features, such as cognitive, behavioral, and motor impairments (Mei et al., 2019; Zuberi et al., 2022). To investigate the underlying disease mechanisms of these both disorders, several GEFS+ and DS mutations have been knocked into the orthologous *Drosophila* gene *para* at corresponding positions (Sun et al., 2012; Schutte et al., 2014; Roemmich et al., 2021). The first variant to be explored was the GEFS+ mutation K1270T in the transmembrane segment 2 of homology domain III (Sun et al., 2012). Since *para* is located on the X chromosome, homozygous female (*para^GEFS+^*/*para^GEFS+^*) and hemizygous male (*para^GEFS+^*/Y) flies were separately assessed. Mutant flies exhibited heat-induced seizures after immersion of the vials containing the flies in a 40°C water bath, regardless of sex. Seizure activity ceased abruptly after removal of the vials from the water bath, i.e., the flies remained motionless and unresponsive for varying time periods. The observed phenotype is reminiscent of the K1270T phenotype in humans, which features febrile seizures. Since GEFS+ is an autosomal-dominant disorder in humans (i.e., patients are heterozygous), heterozygous female flies (*para^GEFS+^*/control) were also assessed. Heterozygous flies exhibited a significantly reduced seizure probability and a delayed seizure onset time compared to homozygous *para^GEFS+^* flies. Also, the seizure activity could start and stop more than once. The cessation of movement upon removal from the water bath was only observed in 46% of the seizing flies. The authors concluded that the heat-induced seizure phenotype of K1270T flies is semi-dominant with variable penetrance. Electrophysiological recordings from GABAergic interneurons of adult mutant flies revealed that the deactivation threshold for persistent currents shifts to a more negative voltage when the temperature is increased. This results in prolonged depolarizations in GABAergic neurons, which causes a decrease in inhibitory activity due to reduced neuronal firing. Thus, it was suggested that the temperature-sensitive seizure phenotype is caused by an overall loss of inhibition (Sun et al., 2012).

A DS-associated *SCN1A* variant that has been explored in *Drosophila* is S1231R. The mutation is located in the transmembrane segment 1 of homology domain III (Schutte et al., 2014). S1231R flies (*para^DS^*) also exhibited a heat-induced seizure phenotype with a significantly increased heat-sensitivity compared with *para^GEFS+^* flies. This aligns with the more severe phenotype of DS observed in humans. However, *para^DS^* flies did not show a cessation of movement upon removal from the water bath. Electrophysiological studies of GABAergic interneurons revealed a constitutional and heat-induced reduction in sodium currents, which resulted in a reduction of repetitive neuronal firing. Besides, the effects of the serotonin precursor 5-hydroxytryptophan (5-HTP) on the seizure phenotypes of *para^GEFS+^* and *para^DS^* flies were investigated. Remarkably, treatment of *para^GEFS+^* flies with 5-HTP caused an increase in seizure probability at high temperatures, whereas treatment of *para^DS^* flies resulted in a suppression of seizures. Electrophysiological studies showed that the treatment did not affect sodium channel properties, but the reduced burst firing frequency was partially rescued. These observations are consistent with the anti-seizure effects of the serotonin releaser fenfluramine in patients with DS (Lagae et al., 2019; Nabbout et al., 2020).

Another study focused on two different *SCN1A* variants that occur at the same position in segment 4 of homology domain IV (Roemmich et al., 2021). While the R1648H (R-H) variant is associated with GEFS+, the R1648C (R-C) variant causes DS. To address the question why different forms of epilepsy arise from mutations at the same amino acid position, *Drosophila* lines carrying either the R-H or R-C mutation were generated by CRISPR/Cas9 gene editing. Flies homozygous for R-H and R-C were lethal, whereas flies heterozygous for these mutations showed spontaneous and heat-induced seizures as well as reduced life spans. Notably, the seizure activity was largely similar between the two mutant strains. Electrophysiological recordings from adult GABAergic neurons showed that both mutations cause sustained depolarizations and reduced firing rates that are exacerbated at elevated temperature. Furthermore, a hyperpolarized deactivation threshold in *para^R-C^* and *para^R-H^* sodium currents was observed, which was present at both room temperature and elevated temperature. Taken together, the similar results from behavioral and electrophysiological studies indicate that the different phenotypes observed in humans may be mainly due to differences in genetic background rather than distinct changes in sodium channel function (Roemmich et al., 2021).

## 3. Comparison of human and *Drosophila* epilepsy genes

### 3.1. Determination of orthologous genes

To study genetic epilepsies in animal models, a robust conservation rate of human disease-associated genes in the respective model organism is a prerequisite. We aimed to compare the orthology of human and *Drosophila* epilepsy genes. Human genes were defined as “epilepsy genes” if they were reported in the review article “Epilepsy-associated genes” by Wang et al. (2017b). To also account for genes identified after the publication date, we also included genes recorded as developmental and epileptic encephalopathy (DEE)-causative genes in Online Mendelian Inheritance in Man (OMIM; https://omim.org/entry/308350) by September 2022 (see full list in Supplementary Table 1). *Drosophila* orthologues of human genes and human orthologues of *Drosophila* genes were defined as genes which had the best score listed on the *Drosophila* online database platform FlyBase^1^ as described previously (Takai et al., 2020). In our paradigm, a minimum score of 6 out 15 possible homology assignments was used as cutoff to define orthologous genes. If two or more genes had identical scores, all genes were counted as orthologues. To define “epilepsy-related” genes in *Drosophila*, a search on FlyBase was conducted using the keyword “epilepsy” and the filters “*D. melanogaster*” and “gene”. All genes listed by September 2022 were considered as “epilepsy-related” genes in *Drosophila* (see full list in Supplementary Table 1).

### 3.2. Conservation rate of human epilepsy genes in *Drosophila*

**Figure 2:**
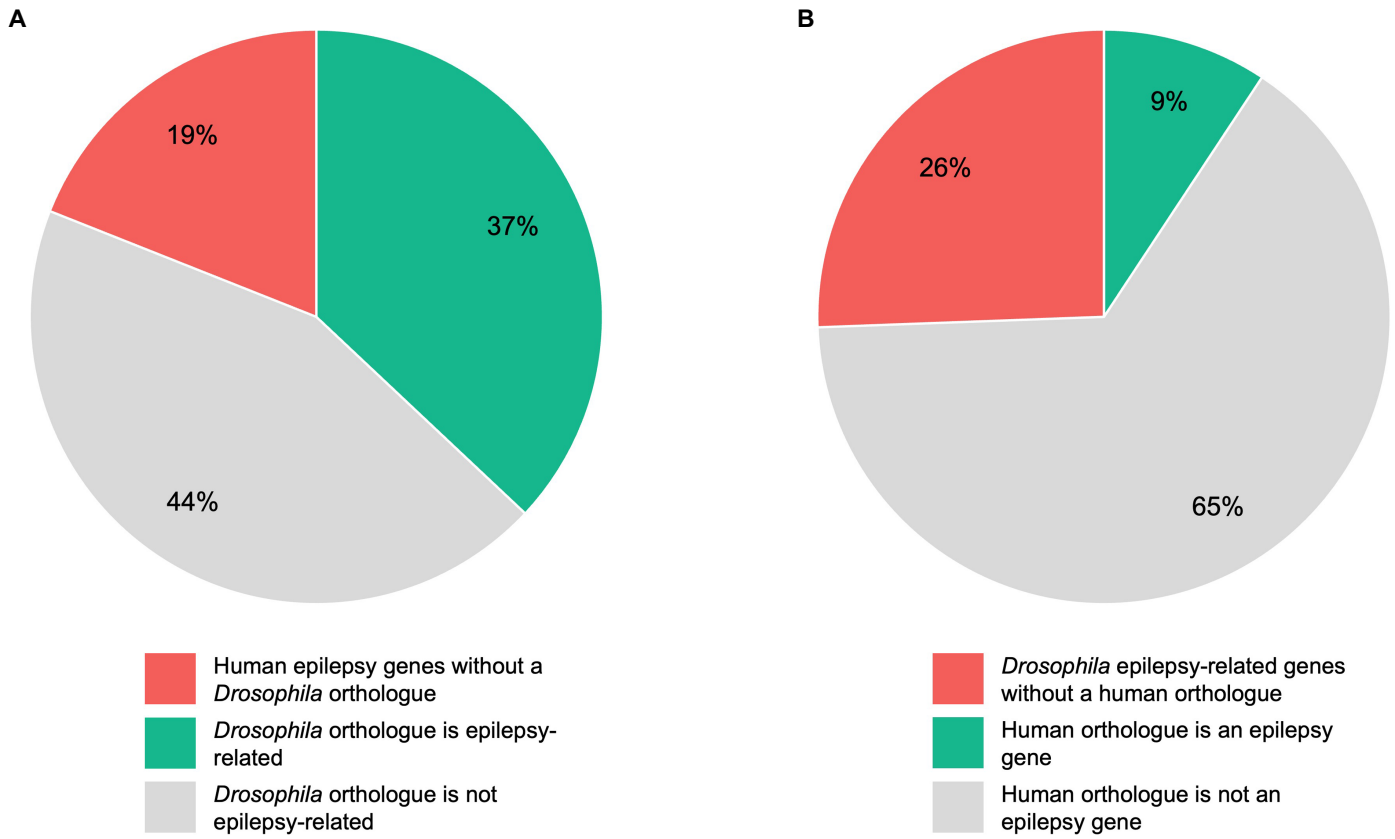
Comparison of human and *Drosophila* epilepsy genes. A total of 145 human genes were defined as “epilepsy genes”, whereas 344 *Drosophila* genes were defined as “epilepsy-related” genes as described under “Determination of orthologous genes”. Pie chart **(A)** shows the 145 human epilepsy genes divided into those genes without a *Drosophila* orthologue, genes whose *Drosophila* orthologue is epilepsy-related, and genes whose *Drosophila* orthologue is not epilepsy-related. Pie chart **(B)** shows the 344 epilepsy-related *Drosophila* genes divided into those genes without a human orthologue, genes whose human orthologue is an epilepsy gene, and genes whose human orthologue is not an epilepsy gene.

To estimate the conservation rate of human epilepsy genes in *Drosophila*, we defined a total of 145 human genes as “epilepsy genes” as described above. Although 75% of human disease-related genes are conserved in flies, we found that this percentage is even higher for genes linked to epilepsy. In detail, we found that 117 of the 145 (81%) human epilepsy genes have an orthologous gene in *Drosophila*, whereas 28 (19%) have no clear orthologue in flies (Figure 2A). To determine which of the orthologous genes are already epilepsy-related in *Drosophila*, we conducted a search on FlyBase. A total of 344 *Drosophila* genes matched our search criteria and were defined as “epilepsy-related” genes. It should be noted that not all of these genes can be defined as “epilepsy-causing” genes as some of the genes are associated with seizure-suppressor mutations, e.g., *topoisomerase 1* (*top1*; Song et al., 2008). According to our search, 145 genes have been linked to epilepsy in humans. Out of these 145 genes, 53 (37%) genes were found to have an epilepsy-related orthologue in *Drosophila*, whereas 64 (44%) genes were found to have a *Drosophila* orthologue that is not epilepsy-related (Figure 2A). Conversely, we aimed to determine how many of the epilepsy-related genes in *Drosophila* have a human orthologue. In *Drosophila*, there are 344 epilepsy-related genes. Out of these 344 genes, 256 (74%) genes were found to have an orthologous gene in humans, whereas no human orthologues were found for 88 (26%) genes (Figure 2B). Of the 344 epilepsy-related *Drosophila* genes, 32 (9%) genes were found to have a human epilepsy gene as an orthologue, whereas 224 (65%) genes were found to have a human orthologue that is not an epilepsy gene (Figure 2B). A list of epilepsy-related *Drosophila* genes and their human orthologues including OMIM numbers (if the human orthologue is an epilepsy gene) can be found in Supplementary Table 2.

### 3.3. Prediction of novel human epilepsy genes using the *Drosophila* model

Since there are 224 epilepsy-related *Drosophila* genes whose human orthologues are not defined as epilepsy genes, it is conceivable that some of these human orthologues could be involved in human epilepsy. Hence, we screened for human genes that are orthologous to more than one of these 224 epilepsy-related *Drosophila* genes since a higher number of epilepsy-related *Drosophila* orthologues may indicate a higher risk of the respective human gene to be associated with epilepsy. Indeed, we found several human genes that are orthologues to more than one of these 224 epilepsy-related *Drosophila* genes (see full list in Supplementary Table 3). For instance, the human *SLC2A8* gene is orthologous to 16 of these 224 epilepsy-related *Drosophila* genes. *SLC2A8* encodes a facilitative glucose transporter (Adastra et al., 2012) that is expressed in a wide variety of tissues, such as testis, brain, liver, heart, and fat (Carayannopoulos et al., 2000; Ibberson et al., 2000). *SLC2A8* has not been previously associated with epilepsy. Importantly, it should be noted that mutations in another member of the SLC2 family, i.e., *SLC2A1* (encoding the glucose transporter type 1), are associated with a variety of distinct epilepsy syndromes, e.g., early onset absence epilepsy, childhood absence epilepsy, and myoclonic-atonic epilepsy (Hildebrand et al., 2014; Koch and Weber, 2019). However, there are also examples where an association with human epilepsy seems rather unlikely. For instance, the human *CPB1* gene, which is orthologous to 8 of these 244 epilepsy-related genes in *Drosophila*, encodes the pancreatic secretory enzyme carboxypeptidase B1. Notably, variants of this gene have been implicated in an increased risk of developing pancreatic cancer (Kawamoto et al., 2022). A link to epilepsy has not been reported yet. Nonetheless, these *Drosophila* genes represent a valuable resource that may help to identify novel human epilepsy genes. Therefore, further analysis of these genes might be a reasonable approach.

## 4. *Drosophila* genetics and tools to investigate seizures in flies

### 4.1. Genetic tools available in *Drosophila*

*Drosophila* as a model organism offers one of the most extensive genetic toolkits available (Senturk and Bellen, 2018) and a nervous system that allows to investigate complex behavior (Ecovoiu et al., 2022). A high conservation rate for human disease-associated genes of ~75% makes it an attractive model to study genetic variants found in humans (Takai et al., 2020). In epilepsy, many severe syndromes are caused by monogenic variants. Information regarding the functional consequences of these variants on the epileptic phenotype is often lacking. As a consequence, the decision on adequate medication can be difficult (Guerrini et al., 2021). Functional analysis of individual disease gene variants in a highly accessible model system is therefore desirable. *Drosophila* offers this possibility in compliance with reasonable labor input and cost. The existing genetic toolkit for *Drosophila* allows in-depth studies of genetic networks and protein function and stock centers all over the world provide many of the necessary fly strains (Bloomington Drosophila Stock Center at https://bdsc.indiana.edu; Kyoto Stock Center at http://www.dgrc.kit.ac.jp; Vienna Stock Center at https://stockcenter.vdrc.at). Generally, two approaches can be taken when assessing disease-related variants in *Drosophila*: Forward-genetics and reverse-genetics (Senturk and Bellen, 2018). In a forward genetic screen, the effects of randomly generated genetic alterations on a specific phenotype are monitored. Unbiased screens for mutations, which either enhance or suppress a certain phenotype (enhancer/suppressor screens) provide a basis for understanding the biological role and interconnection of genes (St Johnston, 2002). They can be used to identify novel therapeutic targets, which might otherwise be overlooked. Screens with chemical agents or transposon-based mutagenesis, which randomly alter parts of the genome, have led to the discovery of several genes associated with seizure-like behavior in flies. Furthermore, screens with these seizure-sensitive flies also enabled the identification of seizure-suppressing variants (Parker et al., 2011a). Reverse genetics seeks to analyze how a phenotype is controlled by a specific genetic sequence. As flies have orthologues for many disease-related human genes, variants first discovered in patients can be investigated for functional aspects in *Drosophila*. Several techniques for introducing these patient-derived variants into the flies are available.

#### 4.1.1. Binary expression systems

**Figure 3:**
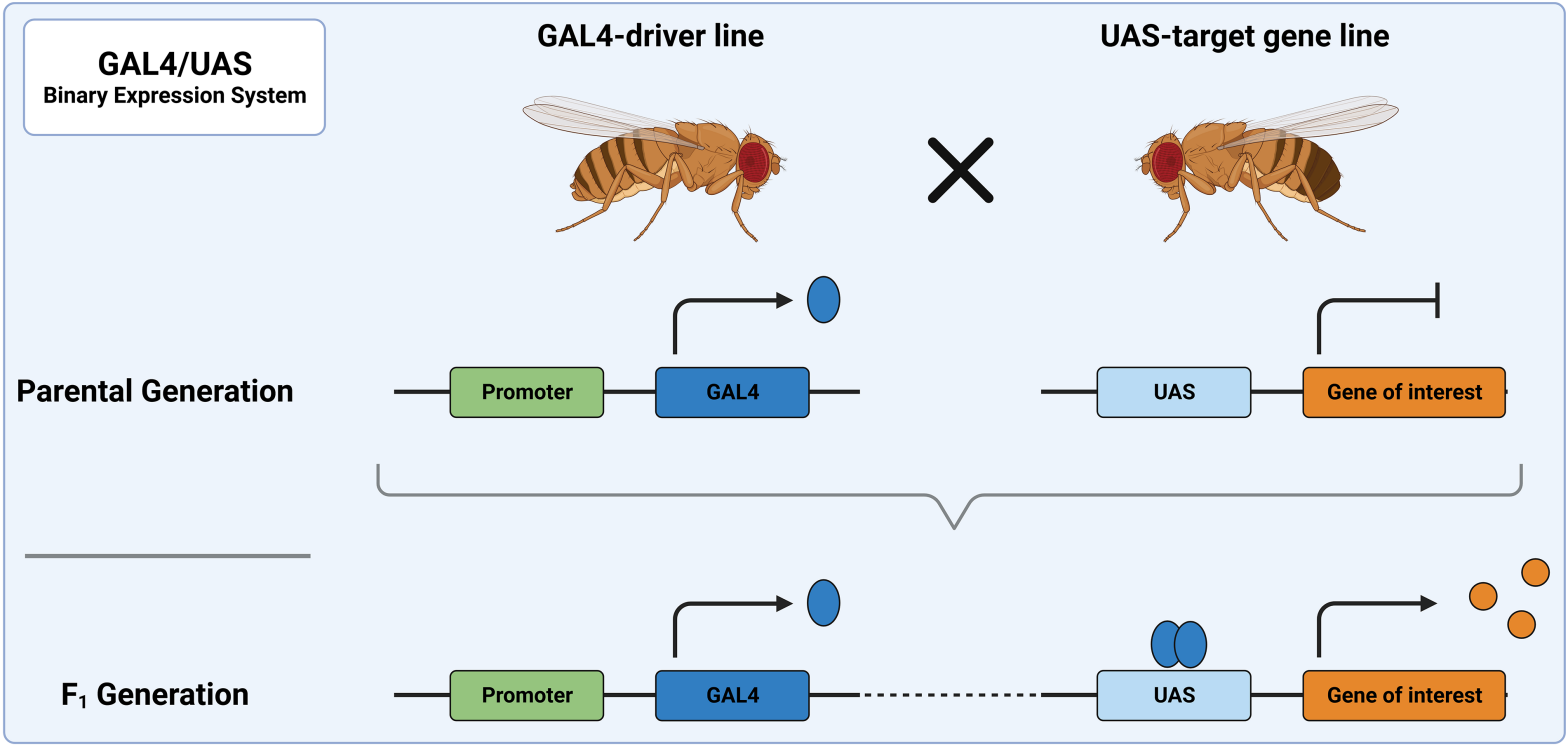
The GAL4/UAS system represents the most widely used binary expression system. In the parental generation, one fly carries GAL4 under a specific promoter and the other a genetic sequence downstream of UAS. When these flies are crossed, the progeny will express GAL4 in all tissues, in which the promoter is utilized. There, it acts as a transcription factor and binds to UAS, leading to expression of the downstream sequence.

Among the primary genetic tools used in *Drosophila* are binary expression systems, e.g., the GAL4/UAS, LexA/LexAop or Q-System (Brand and Perrimon, 1993; Lai and Lee, 2006; Riabinina and Potter, 2016). As the name implies, two components are needed to induce gene expression: a driver of gene expression and a sequence whose expression is controlled by the driver. The driver usually consists of a transcription activator, which is expressed under a tissue-specific promoter. This activator then binds a specific genetic sequence and drives expression of a gene linked to this sequence. In the most frequently used binary expression system, the GAL4/UAS system, expression of a UAS-transgene is achieved through the exogenous (yeast) transcription activator protein GAL4 (Figure 3). The UAS-line harbors a gene/sequence of choice downstream of GAL4 binding sites, called upstream activating sequence (UAS). The UAS-controlled sequence is not expressed in the absence of GAL4. Expression of the UAS-controlled sequence is achieved by crossing a GAL4-driver line with a UAS-line. In the progeny, GAL4 will bind to UAS and facilitate expression of the target sequence downstream of UAS. The GAL4-driver lines were generated as random integration enhancer traps. This way, expression of GAL4 is controlled by an endogenous promoter, which defines the expression domain and allows restricting expression to a region of interest. To date, a plethora of characterized driver lines (> 10,000) are available at aforementioned stock centers and many transgene carrying UAS-lines are available there as well. Some of the most frequently used GAL4-driver lines can be found on FlyBase^3^. Drivers commonly used in the study of the nervous system are, for example, elav-GAL4 or nsyb-GAL4, which allow for pan-neuronal expression of GAL4. Further examples include VGlut-GAL4 and ChAT-GAL4 for confining GAL4 expression to glutamatergic and cholinergic neurons, respectively. The expression of GAL4 follows the expression pattern of the associated gene, which might vary during development of the fly. This should be considered when designing experiments.

Binary expression systems can be used, for example, to overexpress cDNA from a human disease-related gene or the corresponding *Drosophila* orthologue (Jenett et al., 2012; Perkins et al., 2015). This cDNA can either be wild-type or a disease-associated variant. If a disease phenotype is observed after expression of the disease-associated variant, but not after expression of the wild-type, the variant is likely a toxic GOF (Prüssing et al., 2013). This approach has been used to classify *para^bss1^* as a GOF variant (Parker et al., 2011b) or to replicate a severe DEE syndrome by overexpression of *RhoBTB* (Straub et al., 2018). Another possible application of binary expression systems is the induction of RNAi. If a LOF variant is the hypothesis for the disease, RNAi can be used to model the loss of protein function. Expression of inverted repeats (ir) or short hairpins (sh) causes formation of double stranded RNA (dsRNA). If the dsRNA is complementary to endogenous mRNA, this leads to the degradation of target mRNA and a decrease in protein levels (Kaya-Copur and Schnorrer, 2016; Heigwer et al., 2018). The strength of RNAi is influenced by several factors including the levels of interfering RNA, which in turn depends on the activity of the GAL4/UAS system.

To understand the effect of a variant on the phenotype, a rescue experiment can be performed (Ecovoiu et al., 2022). Here, a disease-related phenotype is alleviated by reintroducing a functional allele. For instance, in the case of an epilepsy-related variant in the gene *eas*, expressing wild-type *eas* alleviated the seizure phenotype (Kroll and Tanouye, 2013). Besides already established or newly found epilepsy-related variants in genes, RNAi or deficiency lines can be used as a background for rescue experiments. If a wild-type allele rescues the disease phenotype and a variant does not, this provides compelling evidence for a LOF variant.

Though the GAL4/UAS system is a powerful tool, it requires several considerations. The timing of gene expression is not variable and expression strength is generally elevated at higher temperatures, as GAL4 activity increases. To allow for more precise temporal control over gene expression, inducible GAL4 systems can be employed (Supplementary Figure 1). Additional expression of GAL80 can prevent target gene transcription by inhibition of GAL4 (Suster et al., 2004). Moreover, the temperature-sensitive variant GAL80^ts^ is only active at lower temperatures (Ma and Ptashne, 1987; McGuire et al., 2004; del Valle Rodriguez et al., 2011). This way, temporal control of GAL4 activity is achieved by shifting flies to higher temperatures to inactivate GAL80^ts^. Instead of depending on temperature shifts, the GeneSwitch-GAL4 system depends on the presence of the steroid mifepristone to activate GAL4, which can be added to the standard feeding medium of flies (Osterwalder et al., 2001; del Valle Rodriguez et al., 2011). To allow for better spatial control of GAL4 expression, the split-GAL4 system can be utilized. Here, two different promoters drive expression of a truncated GAL4. Only in cells where both promoters are active, the complementary GAL4 peptides assemble a functional GAL4 (Luan et al., 2020). Even with more specific tools available, these versions of binary expression systems do not fully capture expression profiles of genes of interest, though their easy accessibility makes them useful in initial investigations and for large screens.

#### 4.1.2. Increasing precision of expression

The phenotypic outcome of a given epilepsy-linked variant might be dependent on gene dosage or timing. Here, the mentioned binary expression systems might fall short. To allow better control over expression levels, an endogenous promoter can be hijacked. The transgene can then be expressed in the same pattern of a gene of interest. This can be achieved by utilizing a Minos-mediated integration cassette (MiMIC; Venken et al., 2011). The transposable element *Minos* randomly integrates in the genome and harbors a pair of *attP* landing sites. *Via* the bacteriophage-derived ΦC31 integrase, recombination mediated cassette exchange (RMCE) can be performed. Ultimately, this creates a system with defined integration sites in the *Drosophila* genome, in which a desired genetic sequence can be integrated. Different alterations can be achieved based on the position of the insertion. To drive gene expression, the RMCE site can be placed under an endogenous promoter. By targeting an intron, it can be used to tag a protein with a fluorescent protein. As this system is based on random integrations, the efficiency in creating new lines diminishes with each new line generated.

Besides *Minos*, CRISPR-based strategies (CRIMIC) can also be used to introduce RMCE sites into the genome. Here, integration can be targeted to any position in the genetic sequence accessible to CRISPR/Cas9 modifications. For introns accessible with either method, T2A-GAL4 (“Trojan exon”) can be used to express GAL4 under a promoter of the gene, whose own expression is simultaneously suspended (Diao et al., 2015; Lee et al., 2018). GAL4 can then drive the expression of any UAS construct, while its own expression is controlled by the promoter of the disabled gene. Using this system, different variants can be rapidly screened for their ability to restore protein function, while following endogenous expression patterns. Mutations in the human gene *OGDHL* cause a neurodevelopmental spectrum disease featuring epilepsy. Using T2A-GAL4, gene function could be restored by wild-type expression, but only partially by patient variants introduced into the *Drosophila* orthologue *dOgdh* (Yap et al., 2021). In a similar approach, LOF variants in *TIAM1*, which are associated with seizures, were introduced into *Drosophila* and screened for their ability to rescue a loss of the *Drosophila* orthologue *sif* (Lu et al., 2022). The lower rescuing capacity of patient variants corroborated a LOF effect. As not all genes are suitable targets for CRMIC-based integration of a trojan exon, the gene can also be replaced completely by a GAL4-expressing sequence (Kanca et al., 2022). Large libraries of flies with integration sites in different genes provide broad access to the *Drosophila* genome (Venken et al., 2011; Nagarkar-Jaiswal et al., 2015; Lee et al., 2018).

#### 4.1.3. Direct gene modifications

The most detailed analysis of protein function can be achieved by directly altering the gene itself in the fly genome. In case a human epilepsy-linked mutation is located in a conserved region, the fly gene can be mutated at the respective position to create a humanized variant. Ends-out homologous recombination allows the replacement of the wild-type gene by a mutated variant thereof (Rong and Golic, 2000). Using this technique, patient variants associated with GEFS+ (Sun et al., 2012) or DS (Schutte et al., 2014) have been introduced into the fly genome. To achieve precise genome modifications more efficiently, the CRISPR/Cas9 system has become a widely used tool for genome editing (Jinek et al., 2012; Gratz et al., 2015). In flies, it has been used to introduce patient-specific variants into the gene *para*, which result in either GEFS+ or DS (Roemmich et al., 2021). This allows to study patient-derived variants in a comparable genetic background and to assess, which physiological alterations might be caused by the variants.

Through these methods, genomic modification on different scales becomes feasible for many conserved genes in *Drosophila*. Libraries of fly strains generated by these methods provide researchers access to nearly every gene in *Drosophila*, while direct gene editing remains a possibility to investigate single genes in greater detail.

### 4.2. Techniques for induction and measurement of seizure-like behavior

There are several experimental options to induce seizure-like behaviors in *Drosophila.* For example, flies carrying specific genetic variants are susceptible to mechanical or heat stressors and display seizure-like behavior or paralysis upon exposure to these stressors. The class of bang-sensitive mutants respond to mechanical disturbances (Lee and Wu, 2002) while flies carrying mutations in other genes react to elevated temperatures with seizure-like behavior. Interestingly, some mutant variants only respond to mechanical disturbances or elevated temperature with seizure-like behavior, while others respond to both (Burg and Wu, 2012).

In contrast to rodent models, chemical seizure induction has only rarely been used in *Drosophila*. Treatment with the proconvulsant picrotoxin (PTX) has been shown to induce seizures in larvae (Stilwell et al., 2006). In addition, flies fed with pentylenetetrazole (PTZ) for 7 days displayed a decrease in climbing speed and transcriptomic alterations, which were similar to those reported in human epileptogenesis. Furthermore, an increase in climbing speed was detected 7 days after withdrawal of PTZ (Mohammad et al., 2009). Of note, treatment with ASMs after PTZ withdrawal alleviated the increased climbing speed and normalized PTZ withdrawal-induced transcriptomic changes (Singh et al., 2011).

#### 4.2.1. Techniques for behavioral seizure assays in *Drosophila*

To induce a bang-sensitive phenotype, it has become the standard to use a laboratory “vortex mixer” to shake flies in an empty vial for ~10 s (Kuebler and Tanouye, 2000). If flies have the bang-sensitive phenotype, they display seizure-like behavior and become paralyzed, while wild-type flies remain unaffected. The time until affected flies regain a standing position or mobility correlates with the severity of the phenotype. Administration of ASMs has been shown to reduce the severity of the phenotype in some bang-sensitive mutants (Kuebler and Tanouye, 2002). This reduction could also be achieved by different mutations, which were hence termed seizure suppressors (Saras et al., 2016).

To screen for temperature-related neuronal defects, a reliable way is through a heated water bath, in which flies collected into empty vials can be placed (Burg and Wu, 2012). At a water temperature of ~40°C, seizure-like behavior and paralysis can be observed in susceptible flies as the temperature in the vial increases. Time until seizure or paralysis occurrence, as well as recovery time can be compared. Alterations to seizure susceptibility *via* genetic or pharmacological manipulation can lead to a more heat-resistant phenotype or a faster recovery. In *Drosophila* models of GEFS+ and DS, seizure-like behavior and paralysis occurred at elevated temperatures. A faster onset could be observed in the DS model, corresponding to the increased severity of the variant in patients (Sun et al., 2012; Roemmich et al., 2021). Mutations in other genes can induce non-seizure-related paralysis at elevated temperatures. A notable example is the *shibire* mutant *shi^ts^*, which encodes the *Drosophila* Dynamin orthologue Shibire. At high temperature, the mutant Shibire protein adopts an inactive conformation. As a consequence, there is a block of synaptic vesicle scission, resulting in paralysis (Kosaka and Ikeda, 1983; Hill et al., 2001). A return to lower temperatures allows the mutant Shibire protein to adopt its normal conformation and restores function. Interestingly, overexpression of *shi^ts^* in neurons of bang-sensitive flies leads to a suppression of mechanically induced seizures at an elevated temperature, as endocytosis becomes impaired (Kroll et al., 2015b).

These assays cover only a portion of behavioral assays in *Drosophila*. The behavior of flies can also be assessed in terms of activity, locomotion, memory, or social behaviors (Nichols et al., 2012). For instance, sleep disturbances and changes in locomotor activity were observed in a knock-in model of human GEFS+ (Petruccelli et al., 2015; Mituzaite et al., 2021). Also, flies deficient for *sif* exhibited severe climbing defects besides seizure-susceptibility (Lu et al., 2022). Follow-up investigations of a newfound phenotype with different means are therefore necessary for a comprehensive description.

#### 4.2.2. Investigating neuronal function directly

Electrophysiological recordings allow a more detailed investigation of disturbed neuronal function in *Drosophila*. Seizure-like activity can be induced in all flies, regardless of genetic background, by direct electrostimulation of the brain (Howlett and Tanouye, 2009). To achieve this, electrodes are inserted in both hemispheres of the brain and a short, high-frequent electric stimulus is applied. Depending on the fly’s susceptibility to seizures, different voltages are required to induce a seizure. In bang-sensitive mutants, low voltages are sufficient to induce seizure-like activity, while wild-type or seizure-resistant mutants require higher voltages for seizure induction (Lee and Wu, 2002). Electrophysiological recordings can be performed at the flight or leg muscle. These muscles are innervated by the giant fiber (GF) pathway, an electrophysiologically well-characterized system (Allen et al., 2006). A seizure disturbs GF function and leads to a characteristic display of muscle potentials. While low-intensity single pulse stimulation at the brain is sufficient to activate the GF pathway and create a signal at the innervated muscle, this transmission is abolished during and shortly after a seizure. With this approach, the seizure phenotype of different genotypes can be quantitatively investigated. This includes the voltage needed to induce seizure-like activity, as well as the recovery time, until GF function is restored. Compared to behavioral assays, this method requires a more complex experimental setup and is less suitable for high-throughput applications. To investigate changes in neuronal function in a broader matter, electroretinograms (ERG) can be recorded from the compound eye of the fly (Wu et al., 2022). Here, light-invoked potentials from photoreceptors and neurons are measured at the surface of the compound eye. Changes between measured responses between different genotypes can reflect defects caused by mutations. Besides these methods, patch-clamp experiments at neurons of the adult fly or larvae are possible as a readout for the activity in individual neurons (Marley and Baines, 2011; Sun et al., 2012).

#### 4.2.3. Optogenetic approaches

A non-invasive approach to induce seizures directly at the brain can be taken *via* optogenetic tools. In optogenetics, neuronal function is altered by introducing channelrhodopsins (ChR), which are light-sensitive ion channels (Zhang et al., 2007). Upon stimulation with light of a specific wavelength, the channels open and allow for the flow of anions or cations. This way, neuronal function can be directly controlled. Using the expression systems described above, ChR expression can be targeted to specific areas of the nervous system. Expressing a red-shifted ChR carrying an excitatory current in the brain of several bang-sensitive mutants allows light-activated seizure induction in these flies (Saras et al., 2017). On the other hand, activation of ChR limited to mushroom body neurons was sufficient to induce seizure-like activity in *para^bss1^* flies. This technique offers a way to pinpoint centers of seizure generation in the fly brain. Optogenetic manipulations can not only be utilized for direct seizure induction in adults but also to alter neuronal function of flies during development. DEEs carry a developmental component, which is closely linked to their etiology (Specchio and Curatolo, 2021). A better understanding of variants in disease-causing genes during development will provide a better understanding of disease etiology and allow for a rational design of therapeutic strategies to treat these syndromes. While embryogenesis is challenging to study in mammals, the development of *Drosophila* can be easily observed and manipulated. Expression and activation of ChR pan-neuronally during the development of wild-type flies has been shown to permanently alter network activity and to induce persistent seizure-like activity even in adult flies (Giachello and Baines, 2015). Further limiting these network alterations to specific, critical periods in development has also been sufficient to create this phenotype. Longitudinal administration of ASMs alleviated the seizure phenotype. Besides optogenetic approaches, limiting genetic alterations to these critical periods can lead to a seizure-phenotype as well, highlighting the importance of development-specific effects (Horne et al., 2017).

Besides ChR activation by light, gene expression can also be induced by light (Chan et al., 2015; Lee et al., 2017). Here, photosensitive proteins like cryptochrome are linked to effectors, which drive gene expression following a cascade of interactions after light exposure. As a tool combining light-sensitivity with the GAL4/UAS system, PhotoGal4 offers a light-dependent gene expression system which can be used in conjunction with established UAS-lines (de Mena and Rincon-Limas, 2020). The plant-derived phytochrome B is fused to the GAL4-DNA binding domain (GAL4-DBD), while the phytochrome-interacting factor 6 is fused to a VP16 activator domain (VP16-AD). Exposure to red-light allows for a conformational change of phytochrome B and subsequent binding to the phytochrome-interacting factor 6, which brings together the GAL4-DBD and VP16-AD and induces gene expression. Illumination with light in the far-red spectrum reverts phytochrome B to an inactive form, thereby stopping the interaction and gene expression rapidly. A drawback of this method is the requirement of an exogenous chromophore. Another tool that utilizes light-sensitive proteins fused to a GAL4-DBD or AD is ShineGal4 (di Pietro et al., 2021). Here, DBD and AD are brought together by the interaction of “magnet photoswitches”. These proteins act like magnets and heterodimerize upon exposure to blue light (Supplementary Figure 1). By utilizing the GAL4/UAS system, these systems are “backwards compatible”. This expands the possibilities of investigating gene function at precise points in time during development or in the adult fly.

#### 4.2.4. Detection and visualization of neuronal activity

To further understand network functions and alterations, neuronal activity in flies can be visualized using several strategies (DeNardo and Luo, 2017). Calcium imaging has been extensively used as a readout of neuronal activity (Grienberger and Konnerth, 2012). As calcium is an essential cellular messenger, fluctuations can be directly correlated to neuronal activity and visualized through chemical or genetically encoded calcium indicators (GECIs). Since conventional GECIs require monitoring during behavioral tasks, their use in behavioral applications becomes challenging. For example, loss of the gene *cpes* leads to light-induced seizure behavior, which was also visible as an increase in neuronal activity in a calcium imaging approach in the fly brain (Kunduri et al., 2018). In a simpler setup suitable for larger screens, the calcium-modulated photoactivatable ratiometric integrator (CaMPARI) has been used to label active neurons in freely moving flies (Fosque et al., 2015). CaMPARI is a calcium indicator that uses two components to display neuronal activity. Under conditions where calcium and UV light are available, CaMPARI undergoes a molecular switch and permanently changes from green to red fluorescence. Using UV light only during a defined experimental stimulus, neurons activated in this temporal window can be labeled for functional mapping of neural networks (Zolnik et al., 2017). The technique has been used in *Drosophila* to mark acid-sensing neurons in flies, while they were allowed to move freely in an environment containing either acid or neutralized acid (Edwards et al., 2020). In the context of seizure-like activity, this method might help to further investigate which neuronal networks are crucial during seizure generation. The role of ASMs and other factors modulating seizure-like activity can also be assessed in a visual manner *via* calcium imaging. Preparations of the larval nervous system provided another possibility for large-scale screening of ASMs, as they influenced the spatial–temporal patterns of waves of calcium activity between segments of the ventral nerve chord (Streit et al., 2016).

Other approaches to trace calcium in neurons are based on transcriptional activators. In the presence of calcium, they drive expression of downstream targets like GFP, which then accumulates in recently active neurons. In the CaLexA system (calcium-dependent nuclear import of Lex A), the calcium-responsive transcription factor NFAT (nuclear factor of activated T cells) is fused to LexA and VP16 (Masuyama et al., 2012). The expression of the fusion protein is achieved *via* GAL4/UAS. Presence of calcium leads to dephosphorylation of LexA-VP16-NFAT by the calcium/calmodulin-dependent phosphatase calcineurin and subsequent translocation of the protein to the nucleus, where the LexA domain can drive expression of a reporter. In a similar approach, the TRIC system (transcriptional reporter of intracellular Ca^2+^) utilizes calcium-based binding of calmodulin (CaM) to its target peptide as a reporter of activity (Gao et al., 2015). CaM is fused to a transcriptional activation domain, while its target peptide is fused to a DNA binding domain of a transcription factor like GAL4. Binding of the fusion proteins then facilitates gene expression. The system acts on a slower timescale, reporting activity changes over long periods of time. Both the CaLexA and the TRIC system allow for calcium-based genetic access to active neurons and therefore modulation of those. Similar to optogenetic tools, where disruptions in neuronal activity have already been shown to alter the seizure phenotype under certain conditions (Giachello and Baines, 2015), these tools could be used to directly alter gene function during development.

Overall, *Drosophila* provides a versatile toolkit to induce, quantify and visualize seizure-like behavior. In combination with the vast genetic toolkit, large-scale screens to investigate the role of genes in neuronal function become possible.

### 4.3. Developments in *Drosophila* as a model for epilepsy and future possibilities

**Figure 4:**
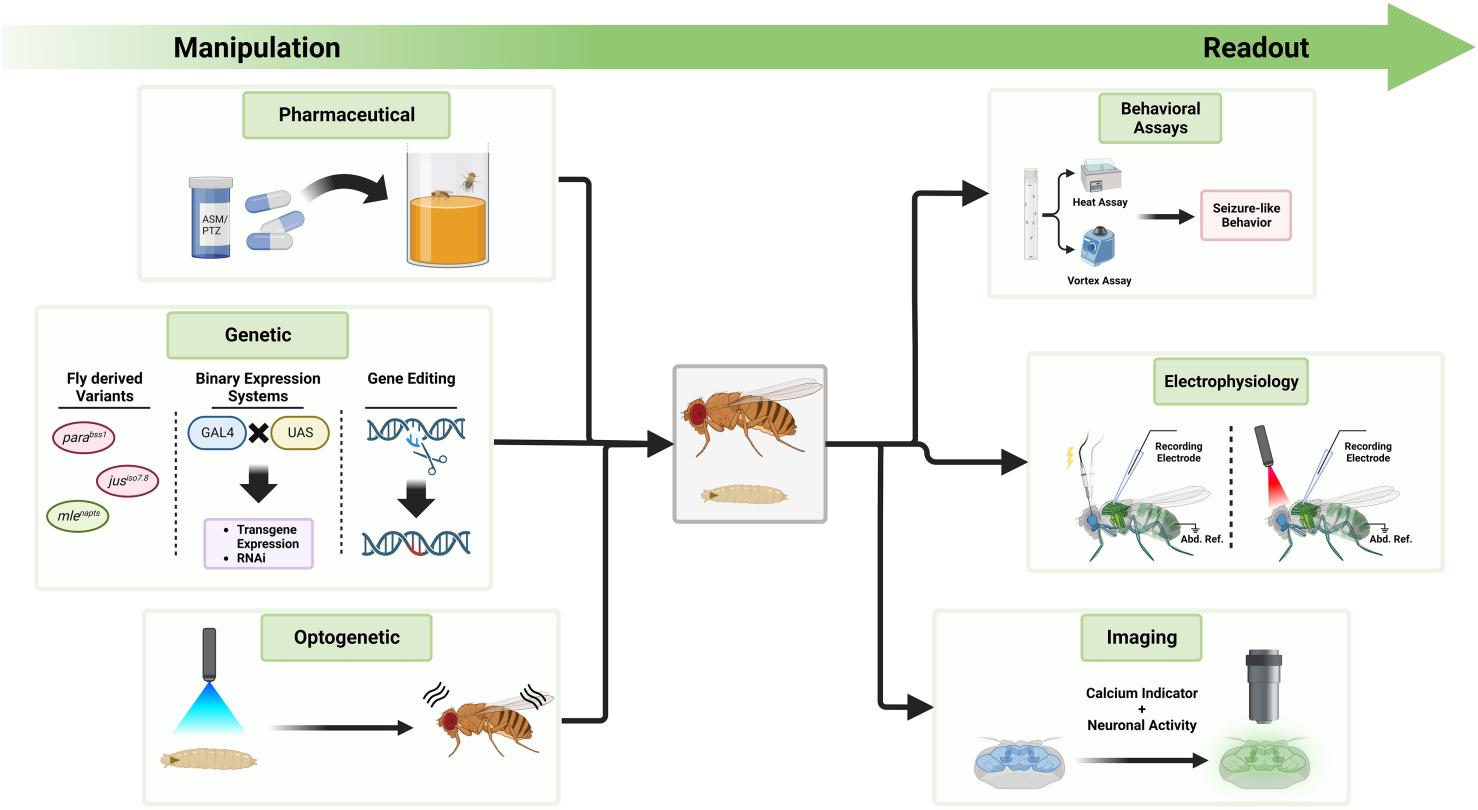
Exemplary tools to manipulate seizure-susceptibility in *Drosophila* and to assess a seizure-like phenotype. The *Drosophila* toolkit allows to manipulate seizure-susceptibility in flies and to assess their phenotype in relation to seizures. On the left side, different methods are displayed through which *Drosophila* can be manipulated. Feeding of compounds like ASMs or PTZ, genetic manipulation, or optogenetic manipulation can exacerbate or decrease seizure-susceptibility. Fly-derived variants increasing seizure-susceptibility are color coded in red, those decreasing it in green. To investigate the effect of these alterations, methods on the right side can be used. These include behavioral assays, electrophysiological measurements and imaging approaches. Seizure induction during electrophysiological measurements can be achieved *via* electrostimulation at the brain or light stimulation if neurons express channelrhodopsins. Activity is measured at the flight muscle with a reference electrode in the abdomen (Abd. Ref.). Techniques on either side can be utilized simultaneously, depending on the research question at hand.

With the extensive methods of genetic manipulation and readouts available, *Drosophila* is a suitable model organism for investigations of genetic epilepsies (Figure 4). Here, we outline modern techniques to investigate seizures in *Drosophila* and their possible applications in the future. Since the number of disease-associated variants of unknown effect size is rising, methods are needed to efficiently study functional consequences of such variants.

#### 4.3.1. Future potential of large-scale screening

Large-scale screens can be performed to study the effects of either genetic variance or compound treatment. Such genetic screens played an important role in forward-genetic approaches in the past, leading to the discovery of many seizure-related genes in *Drosophila* (Parker et al., 2011a). Investigating patient-derived variants of unknown significance on a similar scale is a goal, which becomes now feasible with the genetic toolkit of the fly. Such an approach has already led to the functional description of many epilepsy-related variants in single genes, for example by utilizing T2A-GAL4 technology and UAS-transgenes carrying either wild-type or gene variant for the gene *OGDHL* (Yoon et al., 2017; Yap et al., 2021). The number of epilepsy-related genes and variants will likely increase, and high-throughput screens are needed to meet the demand of functionally testing these variants. In a similar approach to the screen mentioned above, hundreds of variants regarding autism were functionally described in the fly (Marcogliese et al., 2022). Patient variants were expressed under endogenous promoters of fly orthologues or overexpressed in different tissues. These technologies were combined with behavioral monitoring and visualization of gene expression through fluorophores and provided a detailed analysis of different variants. Such a large-scale approach will likely be feasible for variants related to epilepsy as well. Combining such a screen with different methods of seizure induction or suppression might provide valuable information. The recent growth of epilepsy-associated genes also highlighted new molecular pathways and genetic mechanisms. Many of the latest findings have not been functionally explored in *Drosophila* yet (Takai et al., 2020). Investigating rare diseases has also led to a better understanding of common disease etiologies, e.g., in case of neurodegenerative diseases (Ma et al., 2022). This might also become the case for epilepsy caused by non-monogenic reasons.

Many substances have been evaluated for their potential to suppress seizures over the past decades (Loscher et al., 2020). Despite numerous newly marketed ASMs, the proportion of drug-resistant epilepsies is hardly changing (Chen et al., 2018). This unmet clinical need calls for innovative approaches in drug design and drug testing. *Drosophila* has been used for drug-screening before, based on bang-sensitive mutants, in which several anti-seizure drugs showed an ameliorating effect (Pandey and Nichols, 2011). In an RNAi-based approach, differences in expression patterns between wild-type, *para^bss1^* and PTX-fed flies uncovered several potential therapeutical targets (Lin et al., 2017). This allowed to identify and pharmaceutically influence the neuronal homeostasis gene *pumilio*, which was a rather unexpected target (Lin et al., 2018; Mulroe et al., 2022). Another unexpected discovery was that the DNA-topoisomerase I gene *top1* also influences seizure-like phenotypes, emphasizing the potential of broad screens to identify novel therapeutic targets (Song et al., 2007). The rather unexacting approach of candidate compound screening in *Drosophila* also facilitates broader application and is less cost-intensive than in other models. It also allows for exploring less conventional treatments, e.g., agents used in traditional medicine (Dare et al., 2020).

Besides direct effects of drugs or genetic alterations, environmental effects should also be considered when screening for anti-seizure treatments. Though knock-in *Drosophila* models of GEFS+ and DS showed differences in behavior related to the variant severity in patients (Sun et al., 2012), this was not observed to the same degree in CRISPR-derived models for these syndromes, where the amino acid exchange took place at the same site in the protein (Roemmich et al., 2021). The striking differences seen in human variant carriers could not be replicated in flies. Additional genetic factors in patients or fundamental differences between humans and *Drosophila* could explain this observation. Indeed, salient phenotypic variations are not uncommon among individuals carrying identical variants (Helbig and Tayoun, 2016). Recent studies underline that common genetic variants are enriched in persons and families investigated for rare disease-causing variants, hinting at a broad array of influences, that shape a phenotype observed in a patient (Campbell et al., 2022; Oliver et al., 2022). Polygenic mechanisms that influence such epileptic phenotypes might be difficult to study in *Drosophila*, as they are large in numbers with low effect-sizes. Since the genetic background of the fly is very controllable though, decreasing background variation as much as possible might lead to a better understanding of the relationship between rare and common genetic variants. It has also been shown that, similar to possible therapeutic approaches in patients, a ketogenic-like diet can reduce seizure-like activity in several bang-sensitive mutants (Kass et al., 2016; Radlicz et al., 2019). In that regard, controlling living conditions also becomes important and is realized in *Drosophila* by standardizing fly husbandry techniques. In light of these environmental influences, epigenetic mechanisms should be considered as well. Epigenetics describes potentially heritable changes in gene expression that do not involve alterations in the DNA sequence and has become increasingly associated with epilepsy (Van Loo et al., 2022). As epigenetic mechanisms act on wide ranges in the genome, using a genetically well-defined and traceable organism like *Drosophila* might be beneficial to mitigate variance created by such effects.

Certain drawbacks should be considered when using *Drosophila* as a model for drug screening. The composition of fly food used for fly housekeeping or administration of drugs varies considerably between publications (Kruger and Denton, 2020). As not every compound is soluble in water, vehicles like DMSO may be used, which can have an additional effect on the fly. The amount of food consumed by the fly is also difficult to estimate. Colored additives can help with such assessments, as can radioactive tracers or platforms like FlyPAD, which allow for direct tracking of feeding behavior (Deshpande et al., 2014; Itskov et al., 2014; Shell et al., 2018). These methods inevitably increase the labor required to perform feeding experiments though, diminishing one of the advantages of using *Drosophila*. Metabolic processing of a compound and concentrations in different tissues of the fly are not assessed by such methods. The lack of an observable effect of a compound might be a false-negative finding due to these circumstances. Experiments should therefore be performed based on a standardized system. If an effect can be observed, further studies can be designed to solidify the finding.

#### 4.3.2. Gene editing provides cost-effective ways to study patient mutations

Many of the mentioned technologies rely on tools for precise genome editing. CRISPR/Cas9 enables such a precise editing, and the technique has already transformed many areas of biological research, including *Drosophila* research (Port and Bullock, 2016). Through increased utilization of the technique, costs and time needed to generate flies with variants of interest will likely decrease more and more. The amount of patient variants with unclear disease relation rises as a product of advancements in next generation sequencing, and the need for functional studies of these variants rises as well (Bellen et al., 2019). The ability to model variants directly in the *Drosophila* genome is therefore highly desirable. CRISPR/Cas9 provides applications extending the precise genome editing though, such as targeted gene disruption (Port et al., 2020) or to produce UAS-transgene libraries based on human cDNA/ORF (Wei et al., 2020). Direct applications of CRISPR/Cas9-based gene editing in a clinical setting are also discussed, as the possibility to edit dysfunctional genes in somatic cells becomes especially interesting for genes with clear disease relationship (Carpenter and Lignani, 2021). A better understanding of functional aspects of these genes beforehand is critical though and the technology must overcome major obstacles like efficient delivery to targets and prevention of off-target effects, before it becomes clinically relevant.

#### 4.3.3. Precise gene expression and direct control of neuronal function

Recently developed genetic techniques allow for increasingly precise control over gene expression. With CRISPR, a vast number of genes become accessible and can be hijacked or completely replaced to express GAL4 in their place instead (Kanca et al., 2022). For gaining temporal control over gene expression, optogenetic methods like PhotoGal4 or shineGal4 promise light-based on–off-switches, that circumvent the use of less precisely controllable factors like heat or uptake of a compound (de Mena and Rincon-Limas, 2020; di Pietro et al., 2021). Importantly, these methods build on established GAL4/UAS systems, which prevents already established lines from becoming obsolete. Directly controlling neuronal function as opposed to solely controlling gene function provides a valuable asset to the *Drosophila* toolkit as well, especially when investigating neuronal network function. With the ability to genetically target and control specific neuronal subtypes in *Drosophila*, optogenetic approaches promise control over neuronal function at any time (Kohsaka and Nose, 2021). As optogenetics can be used to induce seizure-like activity in *Drosophila* bang-sensitive mutants (Saras et al., 2017), the method will likely gain more traction, being better controllable and less invasive than electrostimulation. However, the established method of electrostimulation enables the assessment of seizure thresholds and can thereby detect subtle changes in seizure susceptibility. Optogenetics, on the other hand, has advantages in precisely targeting specific areas of the nervous system. The complementary use of both methods could help locate sites of seizure generation and determine seizure thresholds. Optogenetic approaches also provide additional ways to study network functions in flies during development (Giachello and Baines, 2015), or to also control gene expression (Chan et al., 2015; Lee et al., 2017). Optogenetics are furthermore researched in clinical settings, although many technical hurdles still have to be overcome before the technology is ready for concrete applications (Wykes et al., 2016).

Derived from the term optogenetics, the term chemogenetics offers similar techniques based on compound effects instead of light. It describes the design of proteins, which interact with small molecules that normally have no physiological relevance (Roth, 2016). In this broad category, Designer Receptors Exclusively Activated by Designer Drugs (DREADDs) gathered the most interest, as they allow for receptor activation through otherwise unrecognized substances. They are often G-protein coupled receptors and activate downstream targets upon activation. DREADDs are employed in epilepsy research and investigated for use in therapeutic settings, similar to optogenetic approaches (Mueller et al., 2022). A clinical application would also require a gene therapeutic approach. This raises concerns about the delivery of receptors, transport of designer agents across the blood–brain barrier and potential off-target effects (Krook-Magnuson and Soltesz, 2015). DREADDs have already been established in *Drosophila* (Becnel et al., 2013) and might provide another tool to alter neuronal functionality during developmental stage or in adult flies. As feeding assays for ASMs are established, the effectiveness of these approaches to suppress seizure-like behavior in flies could be compared. The methods could also be used in concert to evaluate the effects of ASMs while subsets of neurons are controlled through DREADDs or optogenetic approaches.

#### 4.3.4. Databases lay the foundation for future fly research

With the large number of techniques and genes available for studies in *Drosophila*, the amount of generated information becomes difficult to process. Utilizing large data resources and combining different resources will likely play an increasing role. The amount of data generated in terms of patient genomic data, variant effects, structural modeling, network and functional relationships and many more factors becomes ever increasing as well (Lhatoo et al., 2020). With the advent of single-cell RNA sequencing also in *Drosophila*, and extensive connectomes for the fly brain, even larger datasets will become available and combining data in an efficient manner becomes pivotal (Luo et al., 2018; Zheng et al., 2018; Scheffer et al., 2020; Li, 2021). *Drosophila* research provides and relies on large libraries of stocks and curated databases like FlyBase (Gramates et al., 2022), as well as tools to centralize and compare relevant information like MARRVEL (Wang et al., 2017a). Integration of these databases, as well as connectome and other network approaches will play a necessary role to allow for efficient communication in the *Drosophila* field and to make further progress in entangling epilepsy-related mechanisms (Wang et al., 2017a; Mehta et al., 2022).

## 5. Conclusion

The amount of newly discovered genetic variants in patients with epilepsy becomes ever increasing, with many variants remaining functionally uncharacterized. There is a clinical demand to understand the effects of these variants, which is not met by the currently available technologies. Although *Drosophila* has been used as a model organism to study epilepsy for decades, the development of novel technologies and genetic tools has created new possibilities in the field. With the high conservation rate of epilepsy genes, the fly can bridge the gap between other biological model systems, such as mouse and cell culture, and might help to create a better understanding of rare genetic epilepsies. Furthermore, it provides a promising high-throughput platform for the development of novel precision medicine therapeutics.

## Author contributions

All authors listed have made a substantial, direct, and intellectual contribution to the work and approved it for publication.

## Funding

The authors acknowledge financial support from the German Research Foundation (WO 2385/2-1 to SW), the DFG/FNR INTER Research Unit FOR2715 (WE 4896/4-1 and WE 4896/4-2 to YGW), and the Federal Ministry for Education and Research (Treat-ION, 01GM2210B to YGW).

## Conflict of interest

The authors declare that the research was conducted in the absence of any commercial or financial relationships that could be construed as a potential conflict of interest.

## Publisher’s note

All claims expressed in this article are solely those of the authors and do not necessarily represent those of their affiliated organizations, or those of the publisher, the editors and the reviewers. Any product that may be evaluated in this article, or claim that may be made by its manufacturer, is not guaranteed or endorsed by the publisher.

## Figures and Tables

**Table 1:** 

5-HTP	5-Hydroxytryptophan
AD	Activator domain
ASM	Anti-seizure medication
CaLexA	Calcium-dependent nuclear import of LexA
CaM	Calmodulin
CaMPARI	Calcium-modulated photoactivatable ratiometric integrator
cDNA	Complementary DNA
CRIMIC	CRISPR MiMIC
CRISPR	Clustered regularly interspaced short palindromic repeats
DBD	DNA-binding domain
DEE	Developmental and epileptic encephalopathy
DREADDs	Designer receptors exclusively activated by designer drugs
DS	Dravet syndrome
ERG	Electro-retinogram
GECI	Genetically encoded calcium indicator
GEFS+	Generalized epilepsy with febrile seizures plus
GF	Giant-fiber
GOF	Gain-of-function
HDR	Homology-directed repair
LOF	Loss-of-function
MiMIC	Minos mediated integration cassette
NFAT	Nuclear factor of activated T-cells
NHEJ	Non-homologous end joining
ORF	Open reading frame
PTX	Picrotoxin
PTZ	Pentylenetetrazol
RMCE	Recombination mediated cassette exchange
RNAi	RNA interference
TRIC	Transcriptional reporter of intracellular Ca^2+^
UAS	Upstream activating sequence

## Footnotes

^1^ https://flybase.org

^3^ https://flybase.org/GAL4/freq_used_drivers/
