# Supplementary figures and images for: Drosophila melanogaster as a versatile model organism to study genetic epilepsies: An overview
Source: Front Mol Neurosci. 2023 Feb 16;16:1116000. doi: 10.3389/fnmol.2023.1116000 (PMC9978166; doi:10.3389/fnmol.2023.1116000)

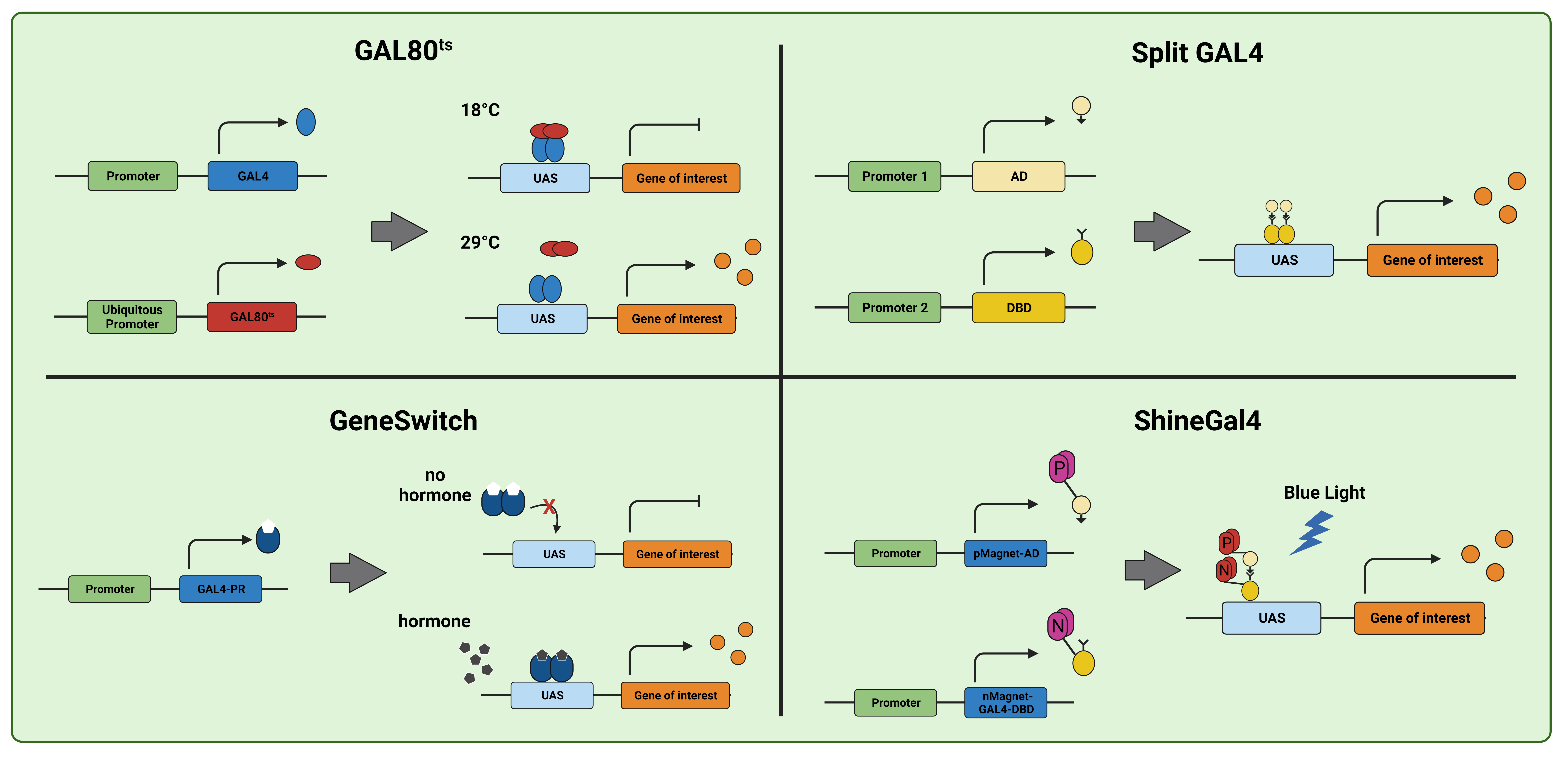

Supplement: Supplementary file 1 [file Image_1.JPEG]
